# A descriptive cross-sectional study on the dry eye disease in children in Xiamen, China

**DOI:** 10.3389/fmed.2026.1897928

**Published:** 2026-08-06

**Authors:** Weifang Fang, Le Sun, Weiwei Xiong, Xue Yin, Hui Yang, Jinghua Bu

**Affiliations:** 1 Department of Ophthalmology, Xiamen Children's Hospital (Children's Hospital of Fudan University at Xiamen), Xiamen Key Laboratory of Pediatric General Surgery Diseases, Xiamen, China; 2 Department of Ophthalmology, Xiang'an Hospital of Xiamen University, Eye Institute of Xiamen University, Fujian Provincial Key Laboratory of Ophthalmology and Visual Science, Fujian Engineering and Research Center of Eye Regenerative Medicine, School of Medicine, Xiamen University, Xiamen, Fujian, China

**Keywords:** CDEQ, COCI, dry eye disease, OSDI, pediatric dry eye disease, questionnaire

## Abstract

**Objective:**

To evaluate the clinical characteristics, risk factors, and applicability of three commonly used dry eye disease (DED) questionnaires (OSDI, CDEQ, and COCI) in children and adolescents in Xiamen, China, aiming to provide preliminary descriptive data in DED pediatric population.

**Methods:**

This single-center cross-sectional study included 105 pediatric patients with DED. Demographic data, clinical findings, risk factors, and questionnaire scores were collected. Univariate analysis was used to identify associated factors. Internal consistency was assessed using Cronbach’s *α*, inter-questionnaire correlations using Spearman analysis, and discriminative validity using Kruskal–Wallis and Mann–Whitney *U* tests.

**Results:**

Among 105 participants (mean age 7.86 ± 3.13 years; 64.76% male), the majority were elementary school-aged (54.29%). All patients exhibited meibomian gland dysfunction, and 92.38% had mixed-type DED. Age (*p* = 0.012), BMI (*p* = 0.067), outdoor activity (*p* = 0.029), and sleep duration (*p* = 0.055) were associated with DED subtype or severity. The response rates of the CDEQ and COCI were 100%. All questionnaires showed acceptable internal consistency (Cronbach’s *α* = 0.642–0.919) and moderate correlations. However, none demonstrated significant discriminative ability for DED severity (*p* > 0.05).

**Conclusion:**

Given the cross-sectional design, no causal inference can be drawn from these findings. Within this descriptive framework, mixed-type DED with predominant MGD emerged as the main subtype in children in Xiamen. Obesity, limited outdoor activity, and short sleep duration were identified as potential correlates. The CDEQ and COCI showed acceptable feasibility and reliability, while the OSDI demonstrated limited applicability. None of the three questionnaires effectively distinguished severity, underscoring the need for pediatric-specific tools.

## 1 Introduction

The Dry Eye Workshop III (DEWS III): Diagnostic Methodology report published by Tear Film and Ocular Surface Society (TFOS) defines dry eye disease (DED) as “a multifactorial, symptomatic disease characterized by a loss of homeostasis of the tear film and/or ocular surface, in which tear film instability and hyperosmolarity, ocular surface inflammation and damage, and neurosensory abnormalities are etiological factors” (1). The global prevalence of DED ranges from 5% to 50%, while epidemiological studies in China have reported a prevalence ranging from 21.0% to 52.4%, with a higher prevalence observed in women and older individuals (2). In recent years, the widespread use of electronic devices and increasing visual demands among school-aged children have contributed to a growing incidence of DED in pediatric and adolescent populations (3, 4). However, current studies have mainly focused on adults, and systematic evidence regarding the clinical characteristics, classification patterns, and risk factors of pediatric DED remains limited.

DED in children is highly heterogeneous, which was related to the immaturity of the ocular surface, variations in lacrimal gland function and various behavioral habits. According to the underlying pathophysiological mechanisms, DED can be broadly classified into evaporative dry eye disease (EDE) and aqueous-deficient dry eye disease (ADDE). EDE is commonly associated with meibomian gland dysfunction (MGD), whereas ADDE results from insufficient tear secretion. Previous studies have suggested that ADDE is relatively uncommon in children, while MGD appears to play a central role in pediatric DED (5). Furthermore, the presence of allergic conjunctivitis or systemic autoimmune diseases is related with ocular surface inflammation (6). Therefore, a systematic analysis of the epidemiological characteristics and lifestyle factors in pediatric DED not only helps understand the onset patterns of DED but also provides a scientific basis for early screening, intervention and individualized management. Furthermore, existing DED questionnaires, such as Ocular Surface Disease Index (OSDI) (7), Chinese Dry Eye Questionnaire (CDEQ) (8) and Chinese version of Ocular Comfort Index (COCI) (9), are primarily designed for adults, and their applicability and reliability in pediatric populations have yet to be validated.

In the single-center study, we conducted a comprehensive cross-sectional analysis of children and adolescents in Xiamen, China, focusing on baseline characteristics, risk factors, and clinical subtypes of DED. In addition, we evaluated the applicability of several commonly used dry eye questionnaires in the pediatric population. Our findings provide valuable evidence for the clinical diagnosis, early prevention, and management of pediatric DED to provide a basis for future multicenter investigations.

## 2 Materials and methods

### 2.1 Study design and ethics

It is a single-center, cross-sectional and observational study that included all children and adolescents (aged ≤18 years) diagnosed with DED who were treated at Xiamen Children’s Hospital between January 1, 2025 and January 30, 2026. The study was approved by the hospital’s Institutional Review Board (Approval No.: 20240617-1). All participants or their guardians signed informed consent forms.

### 2.2 Subject

The inclusion criteria were as follows: According to the TFOS DEWS II diagnostic criteria (7), DED symptoms include persistent dryness, foreign body sensation, and visual fatigue. The clinical signs indicating DED were tear film break-up time (TBUT) ≤ 10 s, Schirmer test (without topical anesthesia) ≤ 10 mm/5 min, and positive corneal fluorescein staining (CFS).

The exclusion criteria were as follows: (1) Use of topical anti-inflammatory or immunosuppressive agents within the past month; (2) congenital ocular surface abnormalities (3) a history of ocular surgery, including punctal occlusion surgery or corneal refractive surgery, within the past 12 months; (4) active ocular infection or allergy; (5) structural eyelid abnormalities (e.g., entropion, ectropion); (6) contact lens use; and (7) any other significant ocular or systemic condition that, in the investigator’s judgment, could interfere with the study assessments or compromise participant safety.

A total of 105 pediatric patients were finally enrolled and divided into three groups according to age: preschool/kindergarten group (2–6 years, *n* = 37, 35.24%); elementary school group (7–13 years, *n* = 57, 54.29%); and middle/high school group (12–15 years, *n* = 11, 10.48%).

### 2.3 Data collection

#### 2.3.1 Basic information

We created a questionnaire to collect basic information, including name, gender, age, height, weight, school grade, mode of delivery and medical history, as well as their lifestyle habits, including dietary habits, duration of close-up activities (such as daily homework and use of electronic devices), duration of outdoor activity and sleep duration about participants.

#### 2.3.2 Questionnaire information

Three questionnaires were used to assess DED in OSDI, CDEQ and COCI. Higher scores indicate more severe DED. The OSDI consists of 12 items across three dimensions: ocular symptoms, visual function, and environmental triggers. Each item is scored from 0 to 4 based on symptom frequency. The OSDI score is calculated as (sum of scores × 25)/number of items answered, yielding a total score ranging from 0 to 100. The CDEQ consists of 12 items from two dimensions: triggers and symptoms. Each item is scored from 0 to 4 based on symptom frequency, with a total score ranging from 0 to 48. The COCI consists of 16 items from eight types of ocular discomfort symptoms and their corresponding severity. Each item is scored from 0 to 10 based on the degree of intolerability, with a total score ranging from 0 to 160.

#### 2.3.3 Clinical signs assessment

We conducted a series of clinical examinations, specifically including the following: visual acuity, intraocular pressure, conjunctival laxity and congestion, tear meniscus height (TMH), TBUT, CFS, Schirmer’s test, palpebral margin assessment (including hyperemia, thickening, neovascularization and MG orifice abnormalities), and MG function (including secretory capacity, meibum quality and gland dropout). All examinations were performed by the same experienced ophthalmologist, and the ocular examination procedures were as follows.

1. Tear Meniscus Height (TMH): As the first non-invasive examination, TMH was measured using a slit lamp with white light. The vertical height of the central lower tear meniscus was observed and recorded. The measurement was repeated three times, and the average value was calculated. A TMH ≤ 0.2 mm was considered abnormal.
2. Tear Film Breakup Time (TBUT): This examination was performed subsequently. A sterile fluorescein sodium strip was gently applied to the conjunctival sac of the lower eyelid to ensure even distribution of the dye. The patient was instructed to blink several times and then to look straight ahead. Under a cobalt blue light with the slit lamp, the time from the last blink to the appearance of the first black spot on the cornea was recorded. The measurement was repeated three times, and the average value was used. A TBUT < 10 s was considered indicative of tear film instability.
3. Corneal Fluorescein Staining (CFS): Immediately after the TBUT examination, corneal epithelial staining was observed and recorded under cobalt blue light. According to the National Eye Institute (NEI) grading scale (10), the cornea was divided into five regions, and based on the density and confluence of staining points each region was scored from 0 to 3: 0 = no staining; 1 = 1–4 scattered punctate dots; 2 = 5–29 dots; 3 = ≥30 dots, confluent staining, coarse dots, or filaments. The total score ranged from 0 to 15.
4. Schirmer’s *I* test: This invasive test was performed last. Without topical anesthesia, a standard Schirmer strip was folded at the 5 mm mark and placed at the junction of the outer one-third of the lower conjunctival sac. The patient was asked to close the eyes gently for 5 min, after which the strip was removed, and the length of wetting was recorded. A wetting length of < 5 mm/5 min was considered diagnostic for DED.

Schirmer’s *I* test was completed in only 28.6% (30/105). The low completion rate was attributed to: (1) poor tolerance to the conjunctival irritation caused by the test strips in younger children (especially those <6 years), and (2) decreased compliance after completion of non-invasive examinations. Accordingly, this data was reported descriptively and was not included in the primary analysis. No imputation was performed for missing data.

According to the *Chinese expert consensus on the diagnosis and treatment of dry eye (2024)* (11), the diagnostic criteria for DED subtypes are as follows (Table 1): ADDE is defined as markedly reduced tear secretion, accompanied by Schirmer’s *I* test ≤ 2 mm/5 min, TBUT > 5 s and TMH ≤ 0.2 mm. Lipid-deficient DED (also EDE) is defined as mildly abnormal tear secretion (Schirmer’s *I* test 5–10 mm), significantly decreased tear film stability (TBUT ≤5 s) and at least three abnormalities of the palpebral margin and MG. Mixed DED is defined as meeting both of the above criteria.

**Table 1:** Diagnostic criteria for different subtypes of DED.

Subtypes	Criteria
Aqueous-deficient DED (ADDE)	Schirmer’s *I* test ≤ 2 mm/5 min, TBUT > 5 s and TMH < 0.2 mm
Lipid-deficient DED (EDE)	Schirmer’s *I* test 5–10 mm, TBUT ≤ 5 s and MGD count ≥ 3
Mixed DED	All of the above

DED severity grades based on TBUT and CFSS (Table 2) (11). Non-DED was defined as TBUT >10 s with CFSS = 0. Mild DED was defined as 5 s < TBUT ≤ 10 s or CFSS = 1. Moderate DED was defined as 2 s ≤ TBUT ≤ 5 s or CFSS = 2. Severe DED was defined as TBUT < 2 s or CFSS = 3.

**Table 2:** Diagnostic criteria for different severities of DED.

Diagnosis	Criteria
Non-DED	TBUT > 10 s and CFSS = 0
Mild DED	5 s < TBUT ≤ 10 s or CFSS = 1
Moderate DED	2 s ≤ TBUT ≤ 5 s or CFSS = 2
Severe DED	TBUT < 2 s or CFSS = 3

### 2.4 Quality control

This study was conducted at Xiamen Children’s Hospital, where a single researcher provided instruction and guidance on questionnaire completion and explained the study purpose. All data were rigorously reviewed, and invalid questionnaires were excluded to ensure data quality.

### 2.5 Statistical analysis

All questionnaire data were first exported to Excel format and then imported into IBM SPSS Statistics 27.0 software for statistical analysis. Continuous variables were first tested for normality. Normally distributed data were presented as mean ± standard deviation (mean ± SD), while non-normally distributed data were presented as median (interquartile range) [*M* (P25, P75)]. Categorical variables were presented as frequencies (percentages) [*n* (%)]. For between-group comparisons of categorical variables, Fisher’s exact test or the *χ*^2^ test was used. Univariate analysis was performed to identify factors associated with DED subtype and severity in children. Independent variables included sex, age, body mass index (BMI), lifestyle habits, and medical history.

Cronbach’s *α* coefficient was used to assess the internal consistency of each questionnaire (*α* > 0.7 acceptable, >0.8 good, and >0.9 excellent). Spearman’s correlation coefficient was used to evaluate correlations among the total scores of the questionnaires. Based on TBUT and CFSS results, participants were classified into mild, moderate, and severe DED groups, and the Kruskal–Wallis *H* test was used to compare questionnaire scores among the three groups. Subsequently, the mild and moderate groups were combined into a non-severe group and compared with the severe group using the Mann–Whitney *U* test.

Our variable selection strategy was as follows (12): potential risk factors were first screened by univariate analysis, and variables with *p* < 0.1 were entered into the multivariate logistic regression model. The threshold of *p* < 0.1 was chosen based on the exploratory nature of this study, to avoid missing potentially important factors given the limited sample size. In the multivariate analysis, *p* < 0.05 was considered statistically significant.

## 3 Results

### 3.1 Characteristics of pediatric patients with DED

A total of 105 children with DED were enrolled in this study, including 68 males (64.76%) and 37 females (35.24%). Baseline characteristics are shown in Table 3. The children had a mean age of 7.86 ± 3.13, height of 130.65 ± 19.80 cm, and weight 29.55 ± 14.37 kg. All participants reported no history of abnormal dietary habits. BMI was calculated using the formula weight (kg)/height (m)^2^, and participants were classified based on the *Weight Management Guidelines (2024 Edition)* issued by the National Health Commission as underweight (*n* = 4, 3.9%), normal (*n* = 82, 78.1%), overweight (*n* = 8, 7.6%), or obese (*n* = 11, 10.5%). According to age, the participants were divided into three groups: preschool (2–6 years, *n* = 37, 35.24%), elementary school (7–12 years, *n* = 57, 54.29%), and secondary school (13–15 years, *n* = 11, 10.48%). The elementary school group had the highest proportion, followed by the preschool group and then the secondary school group, with a statistically significant difference (*p* < 0.001).

**Table 3:** Baseline characteristics of the 105 participants.

Variable	Category	Value (*n* = 105)	Statistical method	*p*-value
Sex	Male	68 (64.76%)	One-sample binomial test	0.003
	Female	37 (35.24%)		
Body type (based on BMI)	Underweight	4 (3.90%)		
	Normal	82 (78.10%)		
	Overweight	8 (7.60%)		
	Obese	11 (10.50%)		
School age and age	Preschool (2–6 years)	37 (35.24%)	One-sample *χ*^2^ test	<0.001
	Elementary school (7–12 years)	57 (54.29%)		
	Second school (13–15 years)	11 (10.48%)		
Mode of delivery	Vaginal delivery; full-term	89 (84.76%)		
	Cesarean section; full-term	14 (13.33%)		
	Cesarean section; preterm	2 (1.90%)		
Daily activities (h/day)	Outdoor activities	1 (0.5, 2)		
	Homework	2 (0, 3)		
	Screen exposure	1.5 (0.6, 3)		
	Sleep	9 (9, 9.75)		

**Figure 1:**
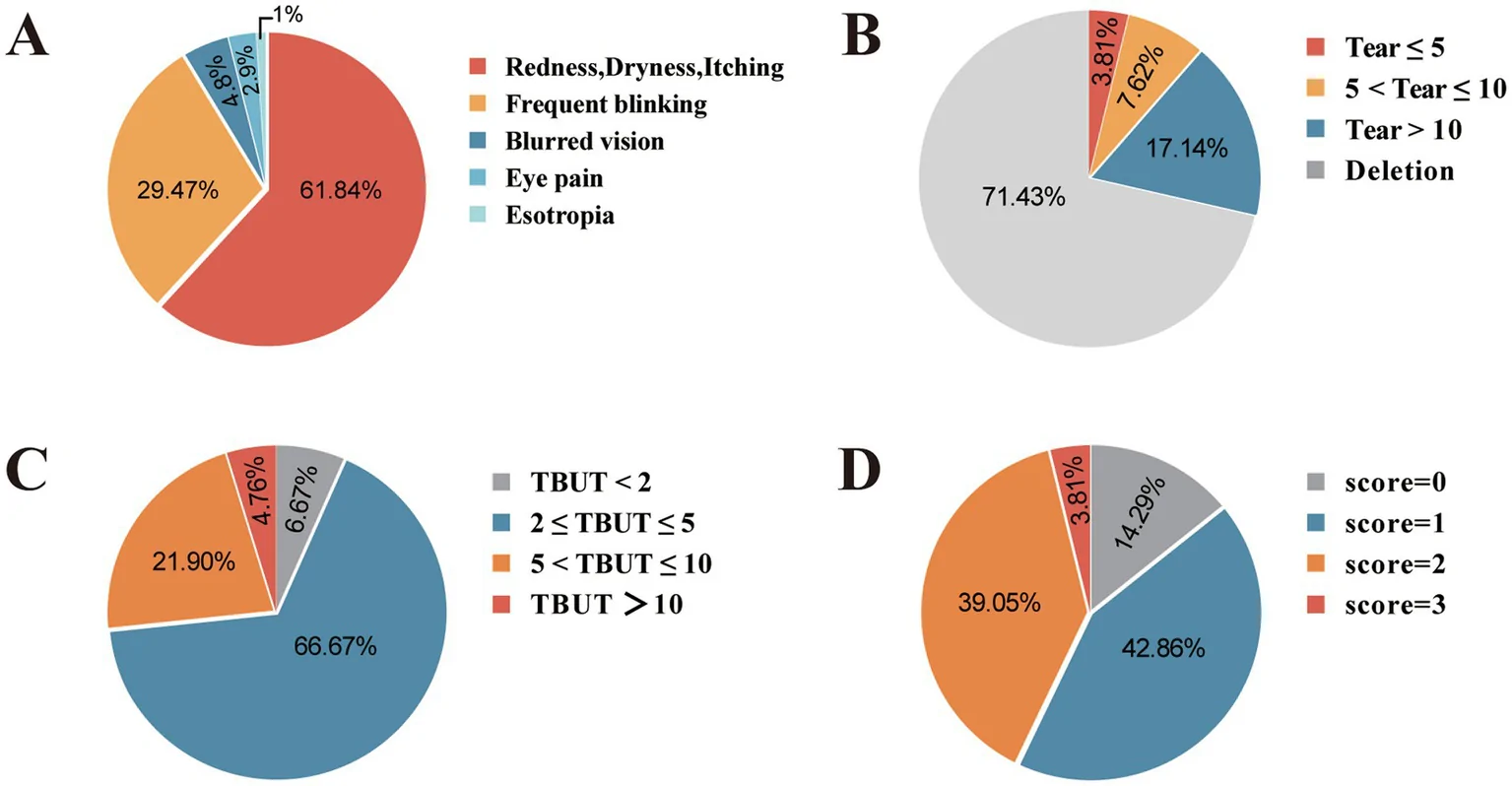
Pie charts showing the distribution of various clinical manifestations of DED among participants. **(A)** Ocular discomfort symptoms; **(B)** Schirmer’s test; **(C)** Tear break-up time (TBUT); **(D)** Corneal fluorescein staining score (CFSS).

Clinical data for participants are presented in Table 4. Shown in Figure 1, the main symptoms among children with DED were redness, dryness, and itching (*n* = 65, 61.90%), followed by frequent blinking (*n* = 31, 29.52%). TBUT was measured in both eyes. Based on the TBUT of the worse eye (the lower value of the two eyes), participants were classified into four groups: TBUT < 2 s (*n* = 7, 6.67%), 2 s ≤ TBUT ≤ 5 s (*n* = 70, 66.67%), 5 s < TBUT ≤10 s (*n* = 23, 21.90%), and TBUT >10 s (*n* = 5, 4.76%). Only 28.57% (*n* = 30) of the children underwent Schirmer’s *I* test, with the following results: tear ≤5 mm/5 min in 4 participants (13.33%), 5 mm/5 min < tear ≤10 mm/5 min in 8 participants (26.67%), and tear >10 mm/5 min in 18 participants (60.00%). Among all participants, 85.71% tested positive for CFS, and 14.29% tested negative.

**Table 4:** DED characteristics of the 105 participants.

Variable	Category		Value (*n* = 105)	
DED questionnaire scores	OSDI		20 (15, 28.87)	
	CDEQ		6 (4, 9)	
	COCI		22 (15, 33)	
Tear film quality	TMH (mm)	<0.2 mm	97 (92.38%)	0.15 ± 0.13^*^
		≥0.2 mm	6 (5.71%)	
	TBUT (s)	<2	7 (6.67%)	4.90 ± 2.96
		2–5	70 (66.67%)	
		5–10	23 (21.90%)	
		>10	5 (4.76%)	
	Tear secretion (mm)		14.68 ± 7.44^**^	
Corneal abnormalities	CFSS	0	15 (14.29%)	
		1	45 (42.86%)	
		2	41 (39.05%)	
		3	4 (3.81%)	
Conjunctival abnormalities	Conjunctivochalasis	Negative	96 (91.43%)	
		Positive	9 (8.57%)	
	Conjunctival hyperemia	Negative	5 (4.76%)	
		Positive	100 (95.24%)	
MG abnormalities	Palpebral margin abnormality	Negative	34 (32.38%)	
		Positive	71 (67.62%)	
	MG orifice abnormality	Negative	30 (28.57%)	
		Positive	75 (71.43%)	
	MG secretion abnormality	Negative	6 (5.71%)	
		Positive	99 (94.29%)	
	MG dropout	Negative	5 (4.76%)	
		Positive	100 (95.24%)	
DED	Subtypes	Aqueous-deficient	0 (0%)	
		Lipid-deficient	6 (5.71%)	
		Mixed	97 (92.38%)	
	Severity	Non	3 (2.86%)	
		Mild	17 (16.19%)	
		Moderate	74 (70.48%)	
		Severe	11 (10.48%)	

^*^N = 103 for TMH measurement; ^**^N = 30 for tear secretion measurement. Data are presented as *M* (P25, P75), mean ± SD, or *n* (%). DED questionnaire scores are presented as *M* (P25, P75); TMH and TBUT are presented as both mean ± SD and *n* (%); tear secretion is presented as mean ± SD; all other variables are presented as *n* (%).

The results showed that all participants exhibited signs of MGD, and most had aqueous deficiency. Assessment of MG revealed palpebral margin abnormality in 67.62% (*n* = 71), MG orifice abnormality in 71.43% (*n* = 75), secretion abnormality in 94.29% (*n* = 99), and MG dropout in 95.24% (*n* = 100) of participants. TMH was measured in 98.10% (*n* = 103) of participants, among whom 92.38% (*n* = 97) had a TMH < 0.2 mm, indicating aqueous deficiency. Based on the subtype criteria, all participants were classified as lipid-deficient DED (*n* = 6, 5.71%) and mixed DED (*n* = 97, 92.38%). Based on the severity criteria, 17 participants (16.19%) had mild DED, 74 (70.48%) had moderate DED, 11 (10.48%) had severe DED, and 3 (2.86%) were classified as non-DED.

### 3.2 Risk factors analysis

Univariate analysis was used to identify factors associated with DED subtype and severity in children (Table 5). The results showed that age (*p* = 0.012), BMI (*p* = 0.067) and outdoor activity (*p* = 0.029) were highly associated with the presence of ADDE. In addition, sleep duration showed a potential association with both the presence of ADDE (*p* = 0.055) and DED severity (*p* = 0.091). Due to the limited sample size (*n* = 105), multivariable regression analysis was not performed to adjust for potential confounders.

**Table 5:** Univariate analysis of risk factors associated with DED.

Variable		*p*-value (subtype)	*p*-value (severity)	Test method
Sex		0.184	0.977	Fisher’s exact test
Age		0.012^*^	0.626	Kruskal–Wallis test
BMI		0.067^*^	0.501	
Lifestyle habits	Screen exposure	0.562	0.850	
	Outdoor activity	0.029^*^	0.571	
	Sleep duration	0.055^*^	0.091^*^	
Medical history	Allergic rhinitis/conjunctivitis	0.902	0.669	
	Myopia	0.466	0.232	
	Epilepsy	0.804	0.683	
	Contact lens	0.664	0.475	
	Ocular surgery	0.724	0.562	
	Antibiotic	0.614	0.407	
	Demodex mite	0.804	0.683	

^*^p < 0.1 indicates a strong association with DED subtype or severity.

### 3.3 Comparison of questionnaires in pediatric DED

#### 3.3.1 Questionnaire response rates

The OSDI consists of 12 items. The response rates for items 1–5 were 100.00%, while those for items 6–12 were 83.81, 0.00, 4.76, 59.05, 70.48, 67.62, and 80.00%, respectively. For both the CDEQ (13 items) and the COCI (16 items), the response rate was 100.00% for all items.

#### 3.3.2 Comparison of questionnaire scores

The results showed that OSDI score was 20 (15, 28.87), CDEQ score was 6 (4, 9), and COCI score was 22 (15, 33) (Table 4). According to the OSDI diagnostic criteria, 91.4% (*n* = 96) of the participants had an OSDI score > 13, indicating the presence of symptomatic dry eye disease in the majority of children.

#### 3.3.3 Internal consistency of the questionnaires

The internal consistency of each questionnaire was assessed using Cronbach’s *α* coefficient (Table 6). The OSDI showed acceptable internal consistency (*α* = 0.747), the CDEQ showed marginal internal consistency (*α* = 0.642), and the COCI demonstrated excellent internal consistency (*α* = 0.919).

**Table 6:** Internal consistency of the questionnaires.

Questionnaire	Number of items	Cronbach’ *α*
OSDI	12	0.747
CDEQ	13	0.642
COCI	16	0.919

#### 3.3.4 Correlation among the questionnaires

**Figure 2:**
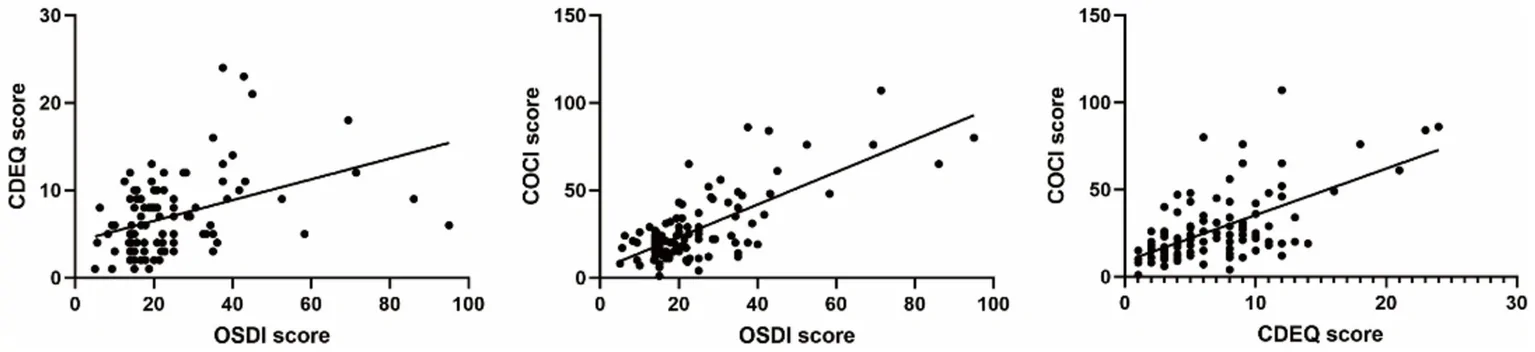
Correlation scatter plots of the three questionnaire scores.

Spearman correlation analysis showed significant positive correlations among the three questionnaires (Figure 2). The correlation coefficient between OSDI and CDEQ was 0.433 (*p* < 0.001), between OSDI and COCI was 0.522 (*p* < 0.001), and between CDEQ and COCI was 0.527 (*p* < 0.001), indicating good consistency of the measured constructs.

#### 3.3.5 Validity of the questionnaires in discriminating DED severity

To evaluate the ability of the three questionnaires to discriminate DED severity in children, participants were classified into mild (*n* = 17), moderate (*n* = 74), and severe (*n* = 11) groups based on TBUT and CFSS. The Kruskal–Wallis *H* test was used to compare the OSDI, CDEQ, and OCI scores among the three groups. As shown in Table 7, no significant differences were observed for any of the questionnaires.

**Table 7:** Validity of the three questionnaires in discriminating DED severity.

Questionnaire	Mild (*n* = 17)	Moderate (*n* = 74)	Severe (*n* = 11)	Kruskal–Wallis test		
				*H*	df	*p*-value
OSDI	21.43 (15, 31.56)	20 (15, 31.04)	20 (14.29, 25)	0.302	2	0.860
CDEQ	5 (4.5, 9.5)	7 (3.75, 10)	5 (4, 8)	0.542	2	0.762
COCI	21 (12.5, 27.5)	22 (15, 34.5)	21 (11, 43)	1.097	2	0.578

*H* = test statistic; df = degrees of freedom; *p*-value = significance.

To further increase statistical power, the mild and moderate groups were combined into a non-severe group (*n* = 91) and compared with the severe group (*n* = 11) using the Mann–Whitney *U* test. Similarly, no significant differences were found for any of the questionnaires (OSDI: *p* = 0.623; CDEQ: *p* = 0.462; COCI: *p* = 0.821). These results suggest that none of the three questionnaires effectively discriminated between different severity levels of pediatric DED in this cohort.

## 4 Discussion

This study systematically evaluated the clinical characteristics, associated risk factors, and the applicability of three commonly used dry eye questionnaires (OSDI, CDEQ, and COCI) in 105 children with DED. Overall, pediatric DED in this cohort was predominantly of the mixed subtype, with a high prevalence of meibomian gland dysfunction (MGD). The most common symptoms included ocular redness, dryness, itching, and frequent blinking. Age, outdoor activity duration, and sleep duration were associated with DED subtype and/or disease severity. Among the three questionnaires, the CDEQ and COCI demonstrated high item response rates, whereas several OSDI items showed poor applicability in children. Although acceptable internal consistency and moderate inter-questionnaire correlations were observed, none of the questionnaires demonstrated sufficient ability to discriminate disease severity.

Pediatric DED was previously considered a rare condition, but its prevalence has increased in recent years (13). Reported prevalence rates range from 6.6% to 20%, with a rate of 18.7% among Chinese individuals under 18 years of age (14). In the present study, elementary school children accounted for 54.29%, and males were slightly more represented (64.76%), consistent with previous findings that pediatric DED is more common in school-aged children with no significant sex difference (15). Overweight and obesity accounted for 18.1%, suggesting an association between abnormal body weight and pediatric DED. Donthineni et al. reported that the main etiologies of pediatric DED were MGD (49%), Stevens–Johnson syndrome (33%), and vitamin A deficiency (9%), with evaporative DED being the predominant cause in adolescents (51%–66%) (16). The present study further showed that mixed-type DED accounted for 92.38%, and the detection rates of MGD were extremely high (dropout 95.24%, secretion abnormality 94.29%, orifice abnormality 71.43%), confirming the central role of MGD in pediatric DED.

In univariate analysis, younger age was associated with shortened TBUT and reduced tear secretion. This trend is consistent with findings from Indian showing a high proportion (76%–96%) of ADDE in infants (16). However, this finding is only from univariate analysis and may be influenced by other factors. A study demonstrated that high BMI was significantly associated with MG tortuosity (OR = 3.79), and suggested a decrease in lipid layer thickness with increasing BMI (17). Although all participants denied abnormal dietary habits, objective measurement revealed that 18.1% were overweight or obese. Experimental evidence has established that high-fat diet induces DED-like ocular surface damage through oxidative stress activation, and impairs both meibomian gland and lacrimal gland function (18–20). More specifically, hyperlipidemia and hyperglycemia have been shown to directly induce MGD (21, 22). These findings highlight the importance of weight management and metabolic screening in the clinical evaluation of pediatric DED. Insufficient outdoor activity was associated with poor tear film stability with reducing blink frequency and increasing ocular surface exposure. A large-scale study involving over 160,000 children found that the prevalence of poor sleep was 80.64% in children with DED, significantly higher than the 64.08% in non-DED children (*p* < 0.001), indicating a close relationship between sleep disturbance and pediatric DED (23).

Questionnaire response rate is an important indicator of its feasibility. In this study, all items of the CDEQ and COCI had a 100% response rate, indicating that both questionnaires were well accepted and understood by children. However, the response rates for items 6–12 of the OSDI were considerably lower, with item 7 (“driving at night”) receiving no response and item 8 (“working on a computer”) answered by only 4.76%, because these items involve several activities that are rarely encountered in children’s daily lives. In addition, cultural factors have influenced the questionnaire responses. Chidi-Egboka et al. noted that some questionnaires contain terminology and language that are difficult for children to recognize (24). The present study suggests that the use of the OSDI in children is severely limited by the applicability of its item content. It is recommended to use only items 1–5 or to develop a modified child-specific version of the questionnaire. Reliability and correlation analyses showed that all questionnaires measured consistent constructs and were able to reflect DED symptoms.

The most important finding of this study is that none of the questionnaires effectively discriminated DED severity in children, in contrast to the well-established ability of the OSDI to reliably differentiate DED severity in adults (25). Possible reasons for the poor discriminant validity include the small sample size of the severe DED group (*n* = 11), differences of expression ability between children and adults, and the limited content validity of the questionnaires. Currently, the severity of pediatric DED can only be assessed through comprehensive clinical tests, as no validated questionnaire is available for this purpose.

This study has several limitations. First, the absence of a non-DED control group prevented the assessment of diagnostic performance, including sensitivity, specificity, and Receiver Operating Characteristic curve (ROC)-based threshold analysis. Second, incomplete data for some examinations, such as the Schirmer’s *I* test (available in only 30 participants), had limited correlation analyses. Finally, this was a single-center study with small sample size (*n* = 105), which limited the generalizability of the findings. Future multicenter studies with larger sample sizes and appropriate control groups are needed to further validate the utility of these questionnaires in pediatric DED and to develop pediatric-specific assessment tools.

## 5 Conclusion

In this cohort, the mixed subtype was the most common form of Pediatric DED, with MGD observed in all cases. Age, outdoor activity duration, and sleep duration showed associations with pediatric DED in univariate analyses. Among the three adult-oriented questionnaires evaluated, the CDEQ and COCI demonstrated good response rates and acceptable reliability in children; however, none of the instruments effectively discriminated disease severity. These findings suggest that currently available adult questionnaires have limited applicability in pediatric populations and highlight the need for the development of validated, child-specific assessment tools for DED.

## Data Availability

The raw data supporting the conclusions of this article will be made available by the authors, without undue reservation.
